# Rotavirus Vaccines Set to Make Inroads in Asia

**DOI:** 10.1093/cid/ciz137

**Published:** 2019-02-13

**Authors:** A Duncan Steele, Umesh D Parashar

**Affiliations:** 1 Enteric and Diarrheal Diseases, Bill & Melinda Gates Foundation, Seattle, Washington; 2 Division of Viral Diseases, Centers for Disease Control and Prevention, Atlanta, Georgia

**(See the Major article by Schwartz et al on pages 2059–70.)**

The implementation of rotavirus vaccines in national immunization programs in ~100 countries (some with phased, subnational introductions) has substantially reduced the disease burden of rotavirus, the leading cause of severe childhood gastroenteritis worldwide [1, 2]. Early introductions in high- and middle-income countries confirmed the large public health impact anticipated based on the high efficacy (85–98%) of the vaccines against severe rotavirus gastroenteritis observed in clinical trials in these settings. Africa has led the implementation of rotavirus vaccines in low-income settings, with nearly three-quarters of African countries routinely vaccinating against rotavirus. Emerging data from many African countries has shown a substantial impact of vaccination on reducing diarrhea hospitalizations and deaths, which is particularly encouraging given concerns about the somewhat moderate rotavirus vaccine efficacy (50–64%) observed in clinical trials in low-income countries [3]. Despite this substantial progress, however, 57% of all children globally still lack access to rotavirus vaccines. In particular, vaccine implementation has lagged in Asia, where less than one-third of countries, including many with large birth cohorts, have implemented national rotavirus vaccination [4]. Additional evidence on the health benefits of rotavirus vaccination from Asian countries will encourage the further adoption of vaccines in the region [5].

In this issue of *Clinical Infectious Diseases*, Schwartz and colleagues report an interesting interrupted time-series analysis of data collected over a 15-year period that examines the population-level impact of rotavirus vaccination in Bangladeshi children. Using data for children residing in villages monitored through a health and demographic surveillance system (HDSS), they examined the impact of rotavirus vaccination administered through a rotavirus vaccine donation program in this HDSS population, following the completion of earlier clinical trials. Because data on both diarrhea hospitalizations and the population under surveillance were accurately captured in the HDSS, these analyses avoid the potential biases from changes in referral patterns or catchment populations that could affect the interpretation of data on the vaccine impact if using hospital-based surveillance alone. Appropriately—given differences in the timing of the vaccine introductions, vaccine coverages, and baseline rates of diarrhea hospitalization, which likely reflect differences in healthcare-seeking behavior and access—they conducted separate analyses for children from villages in icddr,b service areas (ISA) versus government service area (GSA).

There were 2 different time-series models—Model 1, defined *a priori*, and Model 2, defined after an initial examination of the data—used to examine this HDSS data. A comparison of results from the 2 models is complicated by the fact that only a subset of the population used for Model 1 (ie, the population from cluster-randomized villages that did not receive the vaccine in the trial) was used for Model 2. Thus, it is hard to determine to what extent the differences in the results from the 2 models are due to differences in the analytic approaches used, versus differences in the underlying populations. Despite these issues and the post hoc definition of Model 2, the authors present compelling reasons for greater reliance on the results of this model. First, home visits by field staff to encourage treatment for diarrheal episodes during the individually randomized rotavirus vaccine trial conducted during 2007–2009 in the ISA [6] were likely responsible for an increase in the overall healthcare-seeking behavior for diarrhea, since no similar change was seen in the GSA over the same period. If this increased healthcare-seeking behavior was sustained during the later period of routine vaccine use, it would tend to artificially increase the postvaccine rotavirus hospitalization rates and, thus, diminish the measured impact of vaccinations. Secondly, the inclusion of data from control villages for the period during the rotavirus vaccine cluster randomized trial [7] allowed for the inclusion of more contemporary data in the prevaccine baseline and for improved analytic power, because of the availability of 2 additional years of prevaccine baseline data.

Overall, while there were some differences between the results from the 2 models and only the results from Model 2 reached statistical significance, analyses using both models showed a decreasing trend in rotavirus gastroenteritis hospitalizations during the period of routine rotavirus vaccine use, compared with the prevaccine baseline. Several lines of evidence support that this decline was attributable, at least in part, to the rotavirus vaccinations. First, while time-series analyses are susceptible to confounding by other interventions or factors that might affect the incidences of diarrhea if temporally related to the timing of vaccine implementation, the lack of declines in rotavirus-negative gastroenteritis hospitalizations argues against a nonspecific effect. Secondly, greater declines in rotavirus gastroenteritis hospitalization rates were seen in the ISA compared to the GSA, which correlates with the greater rotavirus vaccine coverage achieved during the routine vaccine use period in the ISA versus the GSA. Finally, the observed 39% overall decline in rotavirus hospitalization rates among children <2 years of age in the ISA in the routine vaccine use period is consistent with the decline expected, given the approximately 60–70% vaccination coverage achieved and the 40–60% vaccine efficacy seen in the vaccine trials in Bangladesh and similar settings.

Bangladesh has been a global leader in research documenting the health burden of rotavirus and the potential benefits of vaccination. It is the only country in the world with more than 3 decades of continuous and systematic active surveillance data, coupled with laboratory testing; the latest figures show that approximately two-thirds of childhood diarrhea hospitalizations are attributable to rotavirus [8]. Trials of both the multinational rotavirus vaccines—RotaTeq (Merck and Co) and Rotarix (GlaxoSmithKline)—have been conducted in Bangladesh and have shown efficacy/effectiveness consistent with that in other developing countries [6, 7]. A cost-effectiveness analysis showed that rotavirus vaccination would substantially reduce mortality, illness, and the costs associated with rotavirus vaccine in Bangladesh; would be highly cost-effective if supported through a subsidy from Gavi, the Vaccine Alliance; and can be cost-effective without a vaccine subsidy, depending on the vaccine price [9]. Furthermore, a recent analysis showed that in Bangladesh, where there is limited hospital bed availability and fierce competition for beds, a reduction in rotavirus gastroenteritis inpatients because of vaccination would make more beds available for other patients with childhood morbidities and, indirectly, improve their treatment and outcomes [10]. The analysis by Schwartz and colleagues showing the population-level impacts of rotavirus vaccination further extends and reaffirms the vast evidence of potential benefits from rotavirus vaccination in Bangladesh.

The experience from Bangladesh will also be valuable for policymakers in other Asian countries that have similar rotavirus epidemiologies and burdens in their deliberations around rotavirus vaccine implementation. It is encouraging that the 2 countries with the largest childhood populations in Southeast Asia—India and Pakistan—have both implemented rotavirus vaccination in a phased manner in their national immunization programs over the past 2–3 years. The governments of Bangladesh and Nepal have also recommended national rotavirus vaccination, and these countries have been approved for funding support from Gavi, the Vaccine Alliance, for vaccine purchases. However, the implementation of rotavirus vaccination has been delayed by global supply shortages for both the rotavirus vaccines from the multinational companies—RotaTeq (Merck, West Point, PA) and Rotarix (GlaxoSmithKline, Rixensart, Belgium). Promisingly, 2 new rotavirus vaccines, manufactured in India—ROTAVAC (Bharat Biotech, Hyderabad, India) and RotaSIIL (Serum Institute of India, Pune, India)—were prequalified by the World Health Organization in 2018 and can now be procured with financial support from Gavi, the Vaccine Alliance.

The Indian-made rotavirus vaccines have shown efficacy similar to the multinational rotavirus vaccines in developing countries [11–13], and a recent analysis for 3 low-income countries, including Bangladesh, showed that implementation of the Indian-made rotavirus vaccines will have a substantial public health benefit and that they are highly cost-effective [14]. The availability of multiple cost-effective rotavirus vaccines and the rapidly growing evidence of their public health impact in routine programmatic use should accelerate the implementation of rotavirus vaccines in Asia and globally, thereby achieving the full public health potential of this life-saving intervention.

## Notes

***Disclaimer.***The finding and conclusions in this report are those of the authors and do not necessarily represent the official position of the Bill & Melinda Gates Foundation, nor the US Centers for Disease Control and Prevention.

***Potential conflicts of interest.***Both authors: No reported conflicts of interest. Both authors have submitted the ICMJE Form for Disclosure of Potential Conflicts of Interest. Conflicts that the editors consider relevant to the content of the manuscript have been disclosed.
